# Combination of inositol and alpha lipoic acid in metabolic syndrome-affected women: a randomized placebo-controlled trial

**DOI:** 10.1186/1745-6215-14-273

**Published:** 2013-08-28

**Authors:** Immacolata Capasso, Emanuela Esposito, Nicola Maurea, Maurizio Montella, Anna Crispo, Michelino De Laurentiis, Massimiliano D’Aiuto, Giuseppe Frasci, Gerardo Botti, Maria Grimaldi, Ernesta Cavalcanti, Giuseppe Esposito, Alfredo Fucito, Giuseppe Brillante, Giuseppe D’Aiuto, Gennaro Ciliberto

**Affiliations:** 1 Department of Senology, National Cancer Institute of Naples - Fondazione G. Pascale, Via Mariano Semmola, Naples, 80131, Italy; 2 Department of Division of Cardiology, National Cancer Institute of Naples - Fondazione G. Pascale, Via Mariano Semmola, Naples, 80131, Italy; 3 Division of Epidemiology, National Cancer Institute of Naples - Fondazione G. Pascale, Via Mariano Semmola, Naples, 80131, Italy; 4 Division of Medicine Laboratory and Clinical Pathology, National Cancer Institute of Naples - Fondazione G. Pascale, Via Mariano Semmola, Naples, 80131, Italy; 5 Division of Immunohematology, National Cancer Institute of Naples - Fondazione G. Pascale, Via Mariano Semmola, Naples, 80131, Italy; 6 Division of Pathology, National Cancer Institute of Naples - Fondazione G. Pascale, Via Mariano Semmola, Naples, 80131, Italy

**Keywords:** Metabolic syndrome, Insulin resistance, HDL cholesterol, Inositol, Alpha lipoic acid, Breast cancer

## Abstract

**Background:**

Inositol has been reported to improve insulin sensitivity since it works as a second messenger achieving insulin-like effects on metabolic enzymes. The aim of this study was to evaluate the inositol and alpha lipoic acid combination effectiveness on metabolic syndrome features in postmenopausal women at risk of breast cancer.

**Methods:**

A six-month prospective, randomized placebo-controlled trial was carried out on a total of 155 postmenopausal women affected by metabolic syndrome at risk of breast cancer, the INOSIDEX trial. All women were asked to follow a low-calorie diet and were assigned randomly to daily consumption of a combination of inositol and alpha lipoic acid (77 pts) or placebo (78 pts) for six months. Primary outcomes we wanted to achieve were both reduction of more than 20% of the HOMA-IR index and of triglycerides serum levels. Secondary outcomes expected were both the improvement of high-density lipoprotein cholesterol levels and the reduction of anthropometric features such as body mass index and waist-hip ratio.

**Results:**

A significant HOMA-IR reduction of more than 20% was evidenced in 66.7% (*P* <0.0001) of patients, associated with a serum insulin level decrease in 89.3% (*P* <0.0000). A decrease in triglycerides was evidenced in 43.2% of patients consuming the supplement (*P* <0.0001). An increase in HDL cholesterol (48.6%) was found in the group consuming inositol with respect to the placebo group. A reduction in waist circumference and waist-hip ratio was found in the treated group with respect to the placebo group.

**Conclusions:**

Inositol combined with alpha lipoic acid can be used as a dietary supplement in insulin-resistant patients in order to increase their insulin sensitiveness. Daily consumption of inositol combined with alpha lipoic acid has a significant bearing on metabolic syndrome. As metabolic syndrome is considered a modifiable risk factor of breast tumorigenesis, further studies are required to assess whether inositol combined with alpha lipoic acid can be administered as a dietary supplement in breast cancer primary prevention.

**Trial registration:**

Current Controlled Trial ISRCTN74096908.

## Background

Postmenopausal status tends to increase the risk of being overweight or obese that frequently determines the onset of metabolic syndrome (MS) [1-5]. MS, according to the National Cholesterol Education Program (NCEP) Adult Treatment Panel III (ATP III), can be defined as the presence of at least three of the following clinical criteria: waist circumference >88 cm in women, high-density lipoprotein cholesterol (HDL-C) <50 mg/dl, blood pressure ≥130/85 mmHg, fasting plasma glucose ≥110 mg/dl, or triglyceride >150 mg/dl [6]. MS affects about 34% of the general population. Postmenopausal women are often affected by MS and show the highest incidence of breast cancer in the female population. Breast cancer is also associated with adverse outcomes in patients with metabolic syndrome phenotype [7]. Our previous studies have shown the strict correlation between metabolic syndrome and breast cancer on a huge sample of women [1,2]. Results from those studies reported statistically significant higher prevalence of metabolic syndrome in breast cancer patients with respect to healthy ones (35% vs. 19%) after menopause. Moreover insulin resistance has been reported to greatly contribute to cell growth and breast carcinogenesis [2,8]. These outcomes allowed us to consider MS, and particularly insulin resistance, as modifiable risk factors increasing breast carcinogenesis. Hormonal and metabolic abnormalities may contribute directly or indirectly to an environment for tumor growth. Recently, it has been shown that diabetic patients with breast cancer receiving metformin during neoadjuvant chemotherapy have higher pathologic complete response rates compared with diabetics not receiving metformin [8]. Nevertheless, further studies with larger sample size are needed to assess the validity of novel strategies both for breast cancer chemoprevention and therapy based on targeting insulin signaling pathways. In most of the cases, we have women affected by metabolic syndrome with hyperinsulinemia or insulin resistance but not classical diabetes, and the use of metformin is not allowed for this group of patients. Inositol is a polyalcohol classified as an insulin sensitizer. The phosphatidylinositol 3-kinase (PI3K)/Akt pathway mediates the effects of a variety of extracellular signals in a number of cellular processes including cell growth, proliferation, and survival. The alteration of integrants of this pathway through mutation of its coding genes increases the activation status of the signaling and can thus lead to cellular transformation. The frequent dysregulation of the PI3K/Akt pathway in breast cancer (BC) and the mediation of this pathway in different processes characteristically implicated in tumorigenesis have attracted the interest of this pathway in BC [9,10]. Inositol has been reported to improve insulin sensitivity since it works as a second messenger that may achieve an insulin-like effect on metabolic enzymes [11,12]. Inositol combined with alpha lipoic acid can be used as a dietary supplement in insulin-resistant patients in order to increase their insulin sensitiveness. Inositol is a vitamin B complex constituent that rules as a second messenger in the insulin pathway. Alpha lipoic acid is a fatty acid that plays a leading role in the cellular energetic metabolism exerting antioxidant activities on free radicals, promoting glucose cellular intake, taking part in fat catabolism on the Krebs cycle. Insulin resistance has demonstrated to be a keystone ruling as a gonadotropic and antiapoptotic factor and favoring proinflammatory effects. Furthermore, alteration in lipid profile tends to interfere with cellular growth pattern, promoting cellular damage stimulating free radicals. Alpha lipoic acid strengthens the inositol effects and acts as an antioxidant agent. The combination of inositol and alpha lipoic acid may be a useful supplement to integrate into a regular lifestyle, with a low-calorie diet, physical activity and clinical/radiological examination for postmenopausal women who want to prevent breast cancer and cardiovascular injuries. The aim of the following study was to evaluate the inositol and alpha lipoic acid combination effectiveness on metabolic syndrome features in postmenopausal women at risk of breast cancer by obtaining an optimum control of insulin resistance and lipid profile.

## Methods

### Study design

We carried out a six-month, prospective, randomized placebo-controlled trial on a total of 155 postmenopausal women fulfilling the criteria of metabolic syndrome and at risk of breast cancer (with a familial history of breast or ovarian cancer, or women operated on for borderline lesions such as ductal hyperplasia or papillomatosis). All women were asked to follow a low-calorie diet and were randomly assigned to daily consumption of a combination of inositol and alpha lipoic acid or a placebo for six months.

The distribution of genetic and predisposing risk factors of breast cancer was 60% (93 pts) of patients with a familial history of breast cancer. Some 77% (71 pts) of them had a positive family history of breast cancer in a first-degree relative (mother, daughter, or sister), whereas 23% (22 pts) had a second-degree relative with a familial history of breast cancer. Some 64% (100 pts) of patients had a personal history of previous biopsies for borderline lesions. A total of 95% of women who underwent lumpectomy for borderline lesions had a familial history of breast cancer. Women were invited to attend a ‘high familial risk breast screening program’ set up by the National Cancer Institute. Women aged over forty had a clinical examination, mammogram and ultrasound. Women under forty had a clinical examination, ultrasound and MRI when necessary. Women were examined by the same doctors at all visits.

### Measures and assay methods

In accordance with the Helsinki Declaration of 1975, after obtaining informed consent from each woman, anthropometric features were measured, including weight in kilograms, height in meters, waist and hip circumference, arterial blood pressure was taken and venous blood was collected on study entry. Body mass index (BMI) (kg/m^2^) was calculated from weight and height values and evaluated according to the World Health Organization classification (<25 kg/m^2^ = underweight/normal, ≥25 kg/m^2^ = overweight/obese). The waist-to-hip ratio (WHR) was obtained from waist and hip circumference, measuring the smallest circumference of both to discriminate between android and gynoid fat distribution. Fasting plasma glucose, insulin, HDL-C and triglycerides serum levels were assessed from blood samples. In particular, fasting plasma glucose, HDL-C and triglycerides were measured according to the NCEP ATP III criteria. Blood samples were locally assessed at the central laboratory of the National Cancer Institute. Sample collection was standardized by time at the blood withdrawing. Samples were taken in the early morning hours (between 8.00 and 10.00 am). Fasting plasma glucose assessment was measured by the COBAS INTEGRA Glucose HK cassette (GLUC2; Roche, Basel, Switzerland). It contains an *in vitro* diagnostic reagent system intended for use on COBAS INTEGRA systems for the quantitative determination of the glucose concentration in hemolysate. Electrochemiluminescence immunoassay (ECLIA) applied on Cobas 6000 was used for insulin concentration measurement. The enzymatic colorimetric test CHOD-POD was employed for cholesterol dosage. The GPO-POD method, based on the enzymatic determination of glycerol, using the enzyme glycerol phosphate oxidase (GPO), was used for triglycerides determination. Fresh, clear, unhemolyzed serum was the specimen of choice. The specimen was collected following the guidelines of NCCLS document H4-A3. Insulin levels were defined in the normal range when between 5 and 25 mcU/ml, whereas concentrations above 25 mcU/ml were considered corresponding to hyperinsulinemia. Insulin resistance was calculated by the homeostasis model assessment ratio-insulin resistance (HOMA-IR). To define women as positive for MS we adopted the Adult Treatment Panel III of the National Cholesterol Education Program criteria. All women presenting at least three of the five criteria described on ATPIII were considered affected by metabolic syndrome.

### Baseline demographics of patients

A personal health questionnaire was assessed in order to measure personal health-related quality of life. Women were asked to give information about their daily alcohol intake, smoking, physical activities and sports. A total of 64% of women denied consuming any sort of alcoholic drink. Likewise 64% of women did not smoke cigarettes. On the other hand, only 17% of women answered that they did any physical activity and only 14% declared doing some sports.

### Enrollment steps

•Patients were invited to take part in the trial by their consultants. Women who agreed to take part in the trial were asked to sign a form. However, signing the form did not mean that they must remain in the trial.

•After clinical examination was confirmed to be negative, a mammogram and ultrasound were performed. Women presenting a positive or suspicious mammogram and/or ultrasound were excluded from the trial.

•Anthropometric measures, arterial blood pressure and venous blood samples were taken

•Patients fulfilling at least three of the five criteria described on NCEP ATPIII (waist circumference (WC) >88 cm, HDL-C <50 mg/dl, blood pressure ≥130/85 mmHg, fasting plasma glucose ≥110 mg/dl, or triglyceride >150 mg/dl) were considered affected by metabolic syndrome. Patients affected by metabolic syndrome were considered eligible for enrollment.

•All women were asked to follow a low-calorie diet and were randomly assigned to daily consumption of a combination of inositol and alpha lipoic acid (77 pts) or a placebo (78 pts) for six months. Patients enrolled were asked to follow the Therapeutic Lifestyle Changes (TLC) Diet according to the National Cholesterol Educational Program (NCEP) guidelines. The TLC eating plan is one that advises less than 7% of calories from saturated fat and less than 200 mg of dietary cholesterol per day. There should be no more than 25 to 35% or less of total daily calories coming from total fat intake. A limit of 2400 mg of day of sodium is recommended. The TLC diet recommends weight maintenance and avoidance of weight gain through caloric homeostasis. Reduction of saturated fat, trans fat, and cholesterol within the diet is recommended. Increased consumption of soluble fiber is requested.

•Sample size (ss) was determined using the following formula [13]

$$\mathrm{ss}=\frac{Z^{2}*\left(p\right)*\left(1-p\right)}{c^{2}}$$

Where:

Z = Z value (for example 1.96 for 95% confidence level)

p = percentage picking a choice, expressed as a decimal (.5 used for sample size needed)

c = confidence interval, expressed as a decimal

$$\frac{1.96^{2}*\left(0.25\right)*\left(1-0.25\right)}{0.075^{2}}$$

### Statistical method

Student’s paired *t* test was used for parametric variables and the Wilcoxon test for nonparametric variables like HOMA in order to analyze values at baseline and at the end point. One-way analysis of variance for parametric variables and the Kruskal-Wallis test for nonparametric variables like HOMA were used in order to compare treatment effects of the two arms. *P* <0.0005 was considered statistically significant.

### Exclusion criteria

The exclusion criteria were as follows: women presenting a positive or suspicious mammogram and/or ultrasound; women not fulfilling at least three of the five criteria of metabolic syndrome; diabetic women taking oral hypoglycemic drugs or insulin injections, diabetes diagnosed after a blood sample was taken in our institution, and patients consuming statins for hypercholesterolemia. As diabetes was considered an exclusion criterion, diabetes was diagnosed on laboratory determinations with fasting plasma glucose assessment ≥126 mg/dl according to American Diabetes Association guidelines [12]. Fasting plasma glucose levels in the range between 110 and 126 mg/dl were considered as hyperglycemia.

### Ethical considerations

The INOSIDEX trial was approved by the National Cancer Institute Ethical Committee, protocol number 23/12 OSS from Register M1/2 - metabolic syndrome, insulinemia, BMI in breast cancer prevention - a monocentric non-profit study from the National Cancer Institute of Naples - principal investigator: Dr. Immacolata Capasso. The primary investigators ensured that the study was conducted with full patient compliance, with protocol regulation conformity as well as international standards on human subject research. The primary investigators ensured compliance with institutional regulations as well as local and national law.

### Follow-up phase

This phase comprised of six-monthly visits. Women were examined by the same doctors at all visits. During each visit, a clinical examination was performed, anthropometric features and arterial pressure were measured, blood samples were taken, dietary questionnaires were compiled to understand patient compliance to the study. Health status and medication changes were recorded and adverse events were asked for.

## Results

### Baseline data

The mean age was 57.71 ± 7.9. At baseline mean BMI (kg/m^2^) was 30.35 ± 5.32, mean waist circumference was 97.15 ± 10 cm. Mean WHR was 0.87 ± 0.06. At baseline there was no difference between the two groups in age, BMI, WC, HOMA-IR and lipid profile.

### Primary outcomes

•Reduction of more than 20% of the HOMA-IR index

•Reduction of triglycerides

### Secondary outcomes

•Improvement of HDL-C levels

•Reduction of anthropometric features such as BMI, WHR

### Data analysis

HOMA-IR value total reduction was detected in 86.7% of patients after six months of treatment with inositol and alpha lipoic acid. As a primary outcome, a significant HOMA-IR reduction of more than 20% between baseline and six-month follow-up was evidenced in 66.7% of patients in the treatment group, and there was a statistically significant effect of treatment compared to placebo on HOMA-IR reduction (*P* <0.0001) (Table 1). Serum insulin level decrease was detected in 89.3% of patients consuming the supplement. Wilcoxon test for nonparametric variables confirmed the statistical significant effect of the treatment (*P* <0.05). Regarding lipid profile, significant increase in HDL-C was measured in 48.6% patients in the group consuming inositol with respect to the placebo group after six months of treatment (mean increasing 6%). Reduction in triglycerides (mean 4.9%) was also evidenced in the treated group in 43.2% pts (*P* <0.0001). A little reduction, nonstatistically significant was observed in HOMA-IR, BMI and WC in the placebo group thanks to the low-calorie diet alone. Good control of metabolic syndrome was helped by diet results with the insulin-sensitizing supplements and the related primary prevention of breast cancer. No adverse effects were registered. Good patient compliance and adherence was obtained. All women enrolled in the trial completed it successfully.

**Table 1:** Anthropometric and metabolic features of the study groups: inositol + alpha lipoic acid (n = 77), placebo (n = 78)

	**Ino + α T0**	**Plac T0**	**Ino + α T6**	**Plac T6**	**Ino + α vs Pla *P *T0**	**Ino + α vs Pla *P *T6**	***P *Ino + α T0 vs T6**	***P *Plac T0 vs T6**
Age	57.71 ± 7.9	58.2 ± 5.6						
BMI, kg/m^2^	30.35 ± 5.3	29.21 ± 4.8	28.46 ± 6.2	28.34 ± 5.7	0.1	0.7	<0.0001	0.002
WC cm	97.15 ± 10	99.23 ± 8	92.34 ± 9	97 ± 7	0.08	0.1	<0.000	0.0001
WHR	0.87 ± 0.06	0.89 ± 0.02	0.79 ± 0.04	0.81 ± 0.03	0.1	<0.0001	<0.000	0.0001
Glucose, mg/dl	106 ± 19.58	107 ± 20.13	95 ± 21.56	102 ± 21.79	0.64	<0.0001	<0.000	0.03
Insulin, mmol/L	15.53 ± 8.08	13.74 ± 7.04	8.51 ± 5.11	11.98 ± 6.88	<0.0001	<0.0001	<0.000	0.08
HOMA-IR	4.06 ± 2.44	4.02 ± 1.61	2.74 ± 1.53	3.99 ± 1.22	0.0001	<0.0001	<0.000	0.01
HDL-C, mg/dl	42.30 ± 13.42	40.46 ± 12.45	48.80 ± 14.79	41.72 ± 15.13	0.8	<0.0001	<0.000	0.9
Chol-tot mg/dl	214.63 ± 42.56	203.08 ± 32.71	202.92 ± 49.72	202.01 ± 30.51	0.2	<0.0001	<0.0001	0.3
Triglycerides, mg/dl	111.18 ± 46	112.23 ± 49	90.37 ± 34.36	109.78 ± 48	<0.0001	<0.0001	<0.0001	0.4

Values are means ± SD. Ino + α, inositol plus alpha lipoic acid; T0, time 0; Plac, placebo; T6, 6 months; *P*, *P* value. BMI, body mass index; WC, waist circumference; WHR, waist-hip ratio; HOMA-IR homeostasis model assessment ratio-insulin resistance; HDL-C, high-density lipoprotein cholesterol; Chol-tot, total cholesterol; SD, standard deviation.

## Discussion

### Clinical considerations

MS, characterized by abdominal obesity, high blood glucose levels, impaired glucose tolerance, dyslipidemia, and hypertension, is associated with increased risk of type 2 diabetes and coronary heart disease. Several studies have examined the association of the individual components of MS with BC; to date, however, no study has assessed MS *per se* in relation to BC risk. Furthermore, previous studies have relied only on baseline assessment of components of the syndrome [5]. Nevertheless, a crucial role is supposed to be played by the altered insulin signaling, occurring in obese/overweight patients, which fuels cancer cell growth, proliferation and survival [2]. Insulin resistance is measured through the HOMA-IR score. The cutoff value to define insulin resistance was HOMA-IR ≥2.50. Higher prevalence of MS (35%) was found among postmenopausal women with BC compared to postmenopausal healthy women (19%) (odds ratio 2.16) in our previous study [2]. Visceral adipose tissue has multiple endocrine, metabolic and immunological functions and has been shown to be central in MS pathogenesis [1,2]. Consistent with this, obesity and insulin resistance are supposed to be modifiable risk factors for BC [14,15]. Inositol has been reported to improve insulin sensitivity since it works as a second messenger that may achieve an insulin-like effect on metabolic enzymes [11,12]. Inositol combined with alpha lipoic acid can be used as a dietary supplement in insulin-resistant patients in order to increase their insulin sensitiveness. After only six months of treatment with inositol and alpha lipoic acid, combined with a low-calorie diet, postmenopausal women at risk of BC showed significant reduction in HOMA-IR score and lipid profile control with respect to the placebo group. The data of the present study seems to indicate that MS, particularly insulin resistance, in the postmenopausal population, may be ameliorated by insulin-sensitizing supplementation. As a secondary outcome, we can consider good metabolic control as a useful means in all hormone-related cancer prevention programs, including BC, among postmenopausal women.

## Conclusions

Interestingly, outcomes from our study show that inositol improves insulin sensitivity since it works as a second messenger achieving an insulin-like effect on metabolic enzymes. Inositol combined with alpha lipoic acid can be used as a dietary supplement in insulin-resistant patients not consuming oral hypoglycemic drugs, in order to increase their insulin sensitiveness. The primary end point of our study was to determine the HOMA-IR reduction of more than 20% in patients consuming inositol with respect to the placebo group. A significant HOMA-IR reduction of more than 20% was evidenced in 66.7% (*P* <0.0001) for the treated group of patients. Serum insulin decreasing levels were detected in 89.3% of patients consuming the supplement with inositol. According to the lipid profile, reduction in triglycerides (−43.2%) was evidenced in the treated group (*P* <0.0001). Significant increase in HDL-C (48.6%) was found in the group consuming inositol with respect to the placebo group. Postmenopausal women affected by MS show the highest incidence of BC in the female population. A certain correlation between MS and BC incidence has been widely highlighted in the worldwide literature and MS is now considered a modifiable risk factor of BC. Based on these findings, postmenopausal women affected by MS can easily consume, without any side effects, inositol and alpha lipoic acid as a daily dietary supplement, combined with a low-calorie diet, to reduce insulin resistance and increase HDL-C. MS control can be considered as a first step forward for BC primary prevention.

## Abbreviations

BC: Breast cancer; BMI: Body mass index; HDL cholesterol: High-density lipoprotein cholesterol; HOMA-IR: Homeostasis model assessment-insulin resistance; LDL cholesterol: Low-density lipoprotein cholesterol; MS: Metabolic syndrome; PI3K: Phosphatidylinositol 3-kinase; WC: Waist circumference; WHR: waist-hip ratio.

## Competing interests

The authors declare that they have no competing interests.

## Authors’ contributions

IC realized the protocol design, EE wrote the draft and edited the manuscript. MM, AC and MG contributed to the statistical design. NM recruited metabolic syndrome-affected women. GDA and GC conceived the study idea, supervised the study design and protocol development. GC presented the trial to the ethics committee. MDA and AF recruited patients for the study. GB selected patients at risk of breast cancer. MDL and GF evaluated patients’ compliance to supplementation. EC and GE took blood samples and analyzed them in the laboratory. GB contributing in data managing and preparing informed consent. All authors read and approved the final manuscript.
